# Supplementary material for: Characteristics, treatment outcomes and factors associated with death among patients with Visceral Leishmaniasis, Uganda, 2019–2024
Source: PLoS Negl Trop Dis. 2026 Feb 26;20(2):e0014032. doi: 10.1371/journal.pntd.0014032 (PMC12956114; doi:10.1371/journal.pntd.0014032)
Supplement: Attachment — Submitted filename: PNTD-D-25-01017_reviewer.pdf [file pntd.0014032.s001.pdf]

PLOS Neglected Tropical Diseases

Characteristics, Treatment Outcomes and Factors Associated with Death among Patients with Visceral Leishmaniasis, Uganda, 2019–2024

--Manuscript Draft--

**Manuscript Number:**	PNTD-D-25-01017
**Full Title:**	Characteristics, Treatment Outcomes and Factors Associated with Death among Patients with Visceral Leishmaniasis, Uganda, 2019–2024
**Short Title:**	Visceral Leishmaniasis Patients in Uganda, 2019–2024
**Article Type:**	Research Article
**Keywords:**	Visceral Leishmaniasis, characteristics, treatment outcomes, case fatality rate, associated factors, Karamoja, Uganda
**Abstract:**	Background<br><br>Background: Visceral leishmaniasis (VL) is a neglected tropical disease (NTD) in several countries including Uganda, where it afflicts mainly the Karamoja Region. This region borders Kenya, where VL is endemic. The World Health Organization targets to eliminate VL as a public health problem by reducing case fatality to <1%, but the current burden of VL is unknown. We described VL patients, their treatment outcomes, and identified factors associated with death in Uganda, from 2019-2024, to check progress towards meeting the country's targets.<br>Methods: We reviewed patient files to abstract secondary data of patients diagnosed with VL from 2019–2024, at the main treatment center in Amudat District, Karamoja region. We abstracted data on socio-demographic and clinical characteristics, including co-morbidities, treatment, and treatment outcomes. We used logistic regression to determine factors associated with death.<br>Results: Among 972 patients, 670 (69%) were male and 742 (76%) were age ≤18 years. Three hundred and seventy-three (38%) were from Kenya, while most (72% [434/599]) Ugandan patients were from Moroto District. The highest number of cases (322) was recorded in 2022, with more Ugandans (80%, [259/322) compared to Kenyans, unlike previous years (2019–2021) when there were more Kenyan patients. There was no identifiable seasonal pattern/variation in the number of cases diagnosed. The commonest symptoms were fever (98%), night sweats (77%), and abdominal swelling (72%). The average duration of sickness was 2.6 months (standard deviation=0.3 months). Severe anemia was common (512/972; 53%), and among the patients tested for co-infections, 175/969 (18%) were co-infected with malaria and 185/593 (31%) with HIV. For most patients, 898 (92%), this was their index episode of VL. Almost all patients [957 (98%)] were cured. and most [743 (76%)] patients were treated with the 1st- line regimen. The case fatality rate declined from 2% in 2020 and 2021 to <1% in 2023 and 2024. Being HIV positive was associated with death (Adjusted odds ratios (AOR) 10, 95% Confidence Intervals (CI) 2.2-50, p=0.003).<br>Conclusion: This study indicates progress towards the elimination of VL while highlighting the significance of cross-border transmission and the importance of screening/treatment of co-infections, especially HIV.
**Additional Information:**	
**Question**	**Response**
**Government Employee**<br><br>Are you or any of the contributing authors an employee of the United States government?<br><br>Manuscripts authored by one or more US	Yes - One or more authors are employees of the U.S. government.

Government employees are not copyrighted, but are licensed under a CC0 Public Domain Dedication, which allows unlimited distribution and reuse of the article for any lawful purpose. This is a legal requirement for US Government employees.<br><br>This will be typeset if the manuscript is accepted for publication.	
**Financial Disclosure**<br><br>Enter a financial disclosure statement that describes the sources of funding for the work included in this submission. Review the submission guidelines for detailed requirements. View published research articles from *PLOS Neglected Tropical Diseases* for specific examples.<br><br>This statement is required for submission and **will appear in the published article** if the submission is accepted. Please make sure it is accurate.<br><br>**Funded studies**<br>Enter a statement with the following details:<br>• Initials of the authors who received each award<br>• Grant numbers awarded to each author<br>• The full name of each funder<br>• URL of each funder website<br>• Did the sponsors or funders play any role in the study design, data collection and analysis, decision to publish, or preparation of the manuscript?<br><br>Did you receive funding for this work?	Yes
Please add funding details.<br>as follow-up to "**Financial Disclosure**<br><br>Enter a financial disclosure statement that describes the sources of funding for the work included in this submission. Review the submission guidelines for detailed requirements. View published research articles from *PLOS Neglected Tropical*	This study was supported by the President's Emergency Plan for AIDS Relief (PEPFAR) through the United States Centers for Disease Control and Prevention Cooperative Agreement number GH001353-01 through Makerere University School of Public Health to the Uganda Public Health Fellowship Program, Ministry of Health. The contents of this manuscript are solely the responsibility of the authors and do not necessarily represent the official views of the US Centers for Disease Control and Prevention and the Department of Health and Human Services, Makerere University School of Public Health, or the Uganda Ministry of Health. However, this funding does not cover publication costs.

Diseases for specific examples.<br><br>This statement is required for submission and **will appear in the published article** if the submission is accepted. Please make sure it is accurate.<br><br>**Funded studies**<br>Enter a statement with the following details:<br>• Initials of the authors who received each award<br>• Grant numbers awarded to each author<br>• The full name of each funder<br>• URL of each funder website<br>• Did the sponsors or funders play any role in the study design, data collection and analysis, decision to publish, or preparation of the manuscript?<br><br>Did you receive funding for this work?"	
Please select the country of your main research funder (please select carefully as in some cases this is used in fee calculation).<br>as follow-up to "**Financial Disclosure**<br><br>Enter a financial disclosure statement that describes the sources of funding for the work included in this submission. Review the submission guidelines for detailed requirements. View published research articles from *PLOS Neglected Tropical Diseases* for specific examples.<br><br>This statement is required for submission and **will appear in the published article** if the submission is accepted. Please make sure it is accurate.	UGANDA - UG

**Funded studies**<br>Enter a statement with the following details:<br>• Initials of the authors who received each award<br>• Grant numbers awarded to each author<br>• The full name of each funder<br>• URL of each funder website<br>• Did the sponsors or funders play any role in the study design, data collection and analysis, decision to publish, or preparation of the manuscript?<br><br>Did you receive funding for this work?"	
**Competing Interests**<br><br>On behalf of all authors, disclose any competing interests that could be perceived to bias this work.<br><br>This statement will be typeset if the manuscript is accepted for publication.<br><br>Review the instructions link below and PLOS NTDs' competing interests policy to determine what information must be disclosed at submission.	The authors declare that they have no competing interests.
**Data Availability**<br><br>Provide a **Data Availability Statement** in the box below. This statement should detail where the data used in this submission can be accessed. This statement will be typeset if the manuscript is accepted for publication.<br><br>Before publication, authors are required to make all data underlying their findings fully available, without restriction. Review our PLOS Data Policy page for detailed information on this policy. Instructions for writing your Data Availability statement can be accessed via the Instructions link below.	The datasets upon which our findings are based belong to the Uganda Public Health Fellowship Program (PHFP) Ministry of Health and are not publicly available,but can be availed upon reasonable request from the corresponding author with permission from the Uganda Public Health Fellowship Program.

Manuscript

Click here to access/download;Manuscript;MS_Visceral Leishmaniasis in Uganda 2019-2024_PLOS.docx

Characteristics, Treatment Outcomes and Factors Associated with Death among Patients with Visceral Leishmaniasis, Uganda, 2019–2024

Authors: Benigna Gabriela Namara[1*], Ivan Ankunda[2],Richard Migisha[1], Benon Kwesiga[1], Lilian Bulage[1], Sandra Nabatanzi[3], Alex Riolexus Ario[1], Alfred Mubangizi[2],Daniel Kadobera[3]

Institution affiliations

[1]Uganda Public Health Fellowship Program, Uganda National Institute of Public Health, Kampala, Uganda

[2]Vector Borne and Neglected Tropical Diseases Division, Ministry of Health, Kampala, Uganda

[3]US Centers for Disease Control and Prevention, Kampala, Uganda

[*]**Correspondence:** Benigna G. Namara: benignamara@uniph.go.ug

Abstract

Background: Visceral leishmaniasis (VL) is a neglected tropical disease (NTD) in several countries including Uganda, where it afflicts mainly the Karamoja Region. This region borders Kenya, where VL is endemic. The World Health Organization targets to eliminate VL as a public health problem by reducing case fatality to <1%, but the current burden of VL is unknown. We described VL patients, their treatment outcomes, and identified factors associated with death in Uganda, from 2019-2024, to check progress towards meeting the country's targets.

Methods: We reviewed patient files to abstract secondary data of patients diagnosed with VL from 2019–2024, at the main treatment center in Amudat District, Karamoja region. We abstracted data on socio-demographic and clinical characteristics, including co-morbidities, treatment, and treatment outcomes. We used logistic regression to determine factors associated with death.

Results: Among 972 patients, 670 (69%) were male and 742 (76%) were age ≤18 years. Three hundred and seventy-three (38%) were from Kenya, while most (72% [434/599]) Ugandan patients were from Moroto District. The highest number of cases (322) was recorded in 2022, with more Ugandans (80%, [259/322) compared to Kenyans, unlike previous years (2019–2021) when there were more Kenyan patients. There was no identifiable seasonal pattern/variation in the number of cases

diagnosed. The commonest symptoms were fever (98%), night sweats (77%), and abdominal swelling (72%). The average duration of sickness was 2.6 months (standard deviation=0.3 months). Severe anemia was common (512/972; 53%), and among the patients tested for co-infections, 175/969 (18%) were co-infected with malaria and 185/593 (31%) with HIV. For most patients, 898 (92%), this was their index episode of VL. Almost all patients [957 (98%)] were cured. and most [743 (76%)] patients were treated with the 1st- line regimen. The case fatality rate declined from 2% in 2020 and 2021 to <1% in 2023 and 2024. Being HIV positive was associated with death (Adjusted odds ratios (AOR) 10, 95% Confidence Intervals (CI) 2.2-50, p=0.003).

Conclusion: This study indicates progress towards the elimination of VL while highlighting the significance of cross-border transmission and the importance of screening/treatment of co-infections, especially HIV.

Author Summary

Visceral leishmaniasis (VL), also known as kala-azar, remains a significant but under-researched public health concern in Uganda, particularly in the semi-arid, cross-border Karamoja region. This retrospective study analyzed individual-level data from 972 patients treated for VL at Amudat Hospital between 2019 and 2024 to characterize patient profiles, treatment outcomes, and factors associated with mortality. Most patients were male children, with over one-third originating from neighboring Kenya, highlighting the cross-border nature of VL transmission. The majority presented with typical VL symptoms—fever, night sweats, abdominal swelling, and anemia—and nearly all were diagnosed using rapid diagnostic tests. HIV co-infection was common (30%) and strongly associated with death, making it the only significant predictor of mortality in multivariate analysis. The overall case fatality rate was low (1%), with most patients completing treatment and being cured, indicating substantial progress toward VL elimination targets. However, findings underscore the need for continued surveillance, improved management of co-infections, and coordinated cross-border interventions to interrupt transmission and achieve national and regional VL elimination goals.

Background

Visceral leishmaniasis (VL), also known as kala-azar, is a neglected tropical disease caused by a protozoan parasite of the genus *Leishmania.* It is spread from human to human or from animal to human through the bite of an infected sand fly of the genus Phlebotomus (1–5). The main cause of VL in Uganda is *Leishmania donovani* and humans are considered the main reservoir for this parasite(6–9). Sandflies are attracted to crowded housing because it is easier to bite people and feed on their blood. In addition, human behaviour such as sleeping outside or on the ground may increase the risk of being bitten by a sand-fly (10). The typical clinical features of the disease include fever, weight loss, hepatosplenomegaly, and anemia (2,11).

The disease remains a significant public health problem in Uganda and is one of the Neglected Tropical Diseases (NTDs) that afflicts mainly the Karamoja region—inhabited mainly by pastoralists—where it was first detected in 1951 (12).This region borders the Turkana and West Pokot regions of Kenya—also pastoralist regions—where VL is highly endemic. Despite a number of interventions, VL has remained endemic in the Karamoja region, and it is thought that cross-border migration between Kenya (where VL is also endemic) and Uganda has contributed to its continuous transmission (13,14).

A historical review of VL published in 2014 indicated that the prevalence was higher among males than females and among the 5-14 year age group, although the current burden is unknown (15). A review of data in 2022, on reported cases from health facilities in Uganda reported an average annual incidence of 4 cases per 1,000,000 nationally but a higher burden (139 to 477 cases per 1,000,000) in Amudat district in the Karamoja region, equivalent to an average of 11 to 40 cases per month, of which only 11% were laboratory confirmed (16).

Reported risk factors for VL include malnutrition, immunosuppression due mainly to HIV infection and genetic susceptibility, low socio-economic status, and treating livestock with insecticide, which is common in the Karamoja region (15). On the other hand, sleeping near animals, owning a mosquito bed net, and knowledge about VL were associated with reduced risks for contracting VL (17). The risk factors identified for in-hospital deaths were age <6 years and age >15 years, concomitant tuberculosis, and liver disease (18). A recent (2022) study using routinely collected surveillance data from the District Health Information System version 2 (DHIS2)

reported an annual case fatality rate (CFR) of 5% (16). However, since 2014, there has been no new information on possible risk factors for death, disease outcomes or treatment outcomes. This information is useful for guiding or improving programmatic interventions aimed at the elimination of the disease.

In Uganda, treatment for leishmaniasis is available mainly in three specific treatment centers in three Karamoja districts (Amudat, which is the main and oldest, Moroto, which has been functional for less than 2 years, and Napak for less than one year) L(19). Treatment of this disease consists of a first-line 17-day drug regimen of sodium stibogluconate (S.S.G) + paromomycin (PM) administered intramuscularly daily or a second-line 14-day regimen of liposomal Amphotericin B administered intravenously, for special categories including pregnant women, VL-HIV co-infection, and failure of first-line. These drugs can have severe side effects, which may affect adherence and treatment outcomes. Without treatment, the disease is invariably fatal (13). Treatment outcomes for VL in Uganda are largely undocumented.

All patients meeting the clinical case definition for VL need to be confirmed serologically with Rapid Diagnostic (Blood Dipstick) Tests (RDTs) or parasitologically with a spleen or bone marrow biopsy followed by microscopic identification of amastigotes on stained slides(20).Diagnosis of VL in Uganda relies mainly on the RDTs which allows for an earlier diagnosis and treatment, and therefore an improved prognosis—Parasitoligal diagnosis in not common.

Uganda targets to eliminate VL as a public health problem by 2030, with 85% of the cases detected, 95% treated, and a <1% CFR (21). This underscores the need to reassess the status of VL in Uganda to date, characterize the patients, and evaluate treatment outcomes, and the factors associated. This would inform the development of targeted interventions—such as enhanced surveillance and early treatment in high-risk districts—towards accelerating achievement of the country’s targets. The most recent study on VL in Uganda (2022) only described the spatial and temporal distribution of VL in Uganda using aggregated data from the DHIS2. This study did not fully characterize these patients or determine their treatment outcomes and associated factors (16).

We described VL patients, their treatment outcomes, and identified the factors associated with death in Uganda during 2019-2024 using individual-level patient data from the main treatment center, Amudat Hospital, in Amudat District in Karamoja Region.

Methods

Study design, setting, and data source: We reviewed and analysed surveillance data of patients with VL at the VL treatment center, Amudat hospital, Amudat district in Uganda during 2019-2024. Amudat hospital was selected because it is the main VL treatment center and has been operational since 2000, while the newer centers in Moroto and Napak have only been operational since 2023. It is located in Amudat district in the Karamoja region, which lies in northeastern Uganda (Figure 1). The district is bordered by Nakapiripirit District to the North, Moroto District to the Northwest, and Kenya to the East. The population is predominantly rural, and the people are largely pastoralists who travel to and from the neighbouring VL endemic regions in Kenya. The region is semi-arid with long dry seasons in which sandflies thrive. The predominant sand-fly vector for VL in this region is *Phlebotomus martini* (3). Because the people in this region are pastoralists, they tend to keep large numbers of livestock within their homesteads, which act as feeding grounds for sandflies and increase the risk of human exposure to sandflies. In addition, Karamoja has one of the highest rates of malnutrition in Uganda, particularly among children, which compromises the immune system, making them more vulnerable to VL (22). Conversely, the region has an HIV prevalence of 2.1%, the lowest in the country (23). Surveillance of VL is passive, based on health facility data on patients diagnosed with VL, data is aggregated, entered, and reported monthly into DHIS2. Individual-level data is kept in the patient files at the facility.

Study variables and data abstraction: Using a Kobocollect tool, we abstracted data on patient counts, socio-demographic characteristics, clinical signs and symptoms, date of diagnosis, date of treatment start, and completion/termination, laboratory results, co-morbidities, treatment regimen, length of admission, and disease outcomes from the patient files. Because some NTDs are targeted for elimination, changes in absolute patient numbers are usually used as a measure of the burden of disease and progress towards elimination(24).

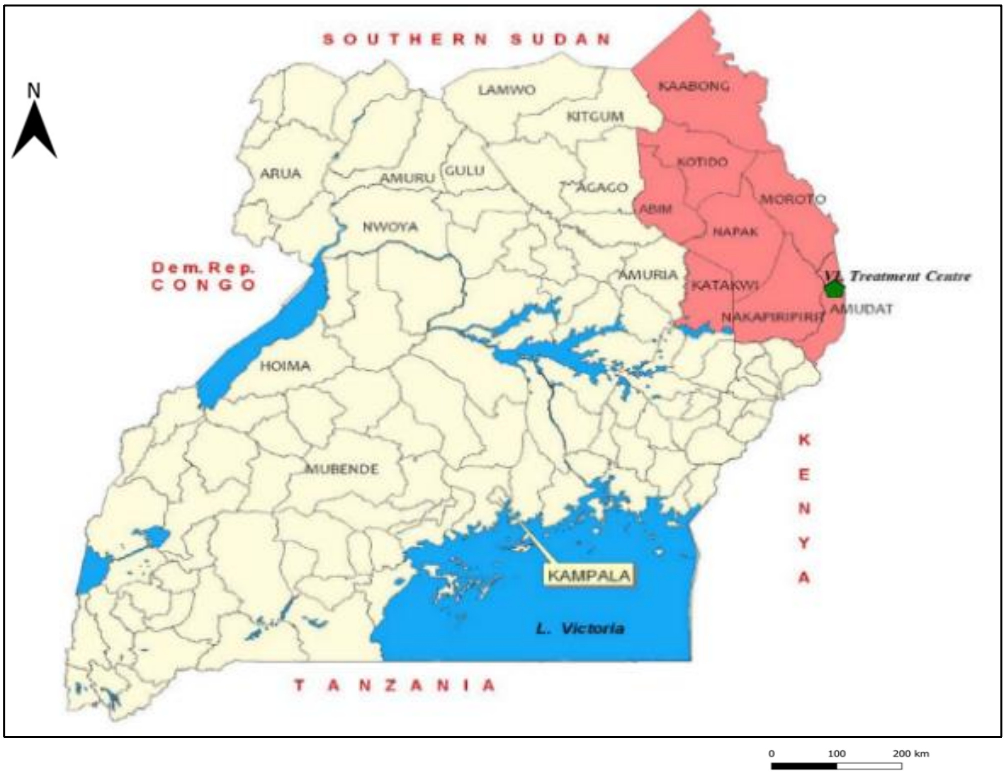

Figure 1: Amudat Visceral leishmaniasis Treatment Center, Karamoja, Uganda: Adapted from Ministry of Health, Uganda

Data analysis: We analyzed the data using Microsoft Excel and Stata version 12.0. We calculated proportions for categorical variables, including patient characteristics such as sex, residence, symptoms, co-morbidities, treatment, and treatment outcomes; and summary statistics for continuous variables such as age and duration of illness using means and standard deviation (SD). We assessed for seasonal variation by comparing the numbers of VL cases in the months of the rainy season with those in the dry season. We employed logistic regression models to identify factors associated with death. The multivariable model was fitted using a bidirectional stepwise elimination approach. Only predictive variables with a significance level of $p \leq 0.2$ at bivariate analysis were included in the model after performing chi-square tests for independence. To assess the model's fit, we used the Hosmer-Lemeshow goodness-of-fit test. Odds ratios (ORs) and their corresponding 95% confidence intervals (CIs) were examined to quantify the strength and direction of association between predictors and death. Cases with missing values for any of the predictor variables were excluded from the analysis.

Results

Demographic characteristics of Visceral Leishmaniasis patients, Uganda, 2019–2024

A total of 972 patient records were reviewed for the period 2019-2024; 670 (69%) were male, the mean (standard deviation[SD]) age was 14(SD 2) years. The majority (61.6%) of VL patients were Ugandan, but 373 (38%) came across the border in Kenya (the majority (38%) of whom lived in the Turkana region). Most (72.5%, n=434) of the Ugandan patients were from Moroto district. The highest number of cases (322) was reported in 2022, with more Ugandans than Kenyans compared to previous years, when there were more Kenyans (Figure 2). There was no identifiable seasonal variation/pattern in the number of cases diagnosed (Figure 3).

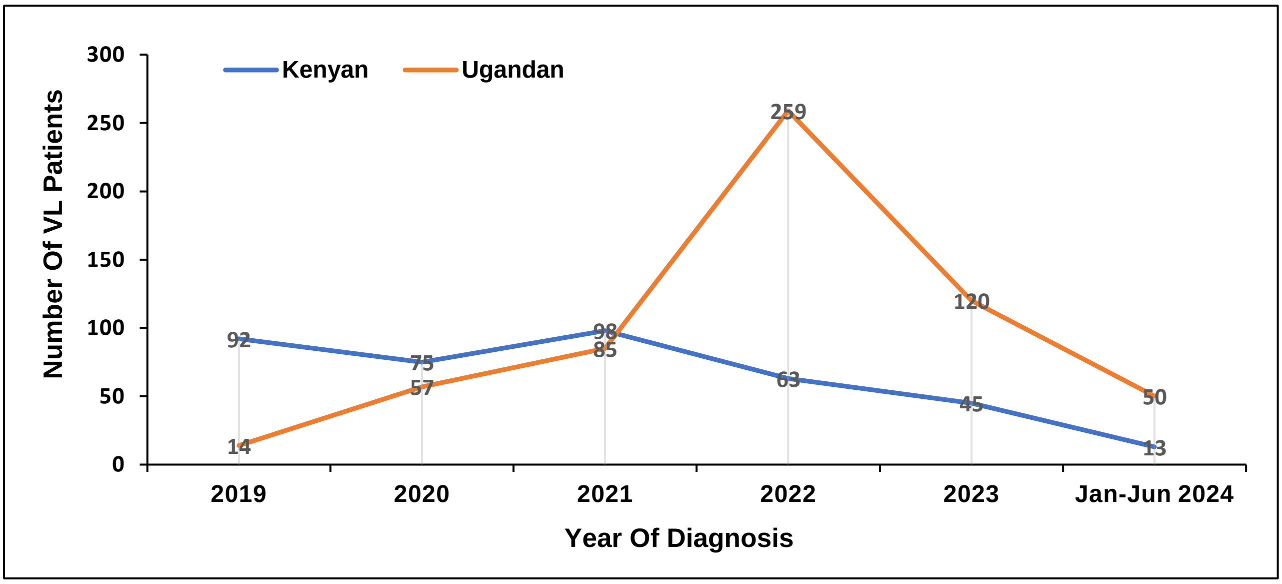

Figure 2: Number of Visceral Leishmaniasis (VL) patients per year, Uganda, 2019–2024

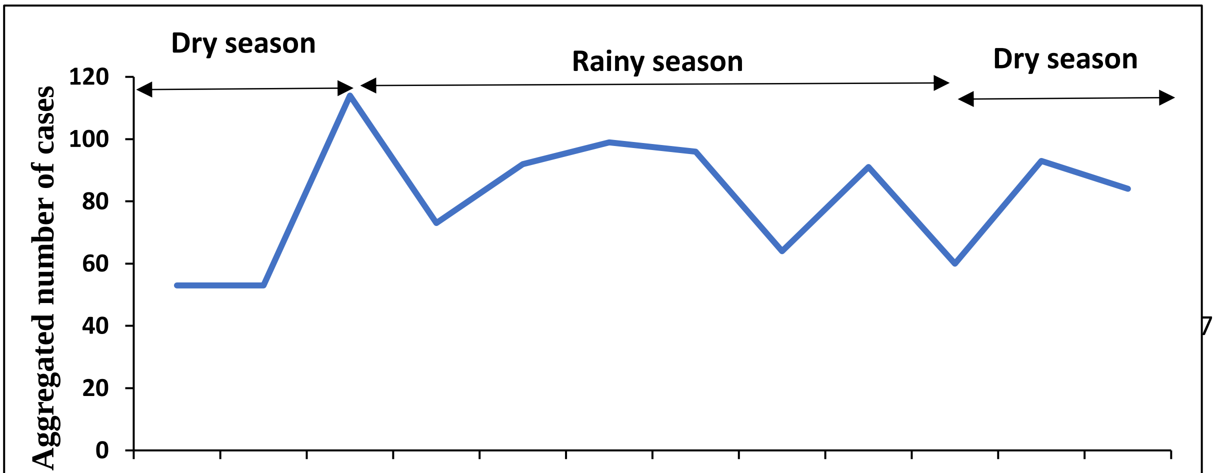

Figure 3: Number of Visceral Leishmaniasis patients per month, Uganda, 2019–2023

Clinical characteristics of Visceral Leishmaniasis patients, Uganda, 2019–2024

Nine hundred and forty-three (97%) of the 972 VL patients were confirmed using the Leishmania dip-stick test. The most common symptoms of VL patients included fever (98%), night sweats (77%), abdominal swelling (72%), and weight loss (68%). The average duration (SD) of sickness was 2.6 (0.3) months. VL patients were tested for co-infections; 177 (18%) had malaria, 287 (30%) tested HIV positive, 2.7% had Hepatitis B. Additionally, 47% had severe anemia.

Treatment characteristics and outcomes of Visceral Leishmaniasis patients, Uganda, 2019–2024

For most (895 [92%]) patients, the VL episode recorded was their index episode; only 8% were having a recurrence. Most patients received the first-line treatment regimen, 229 (24%) were treated with the second-line regimen, and almost all patients were cured; the overall case fatality rate was 1% (Table 1). Among patients on second-line treatment, 179 (78%) were HIV positive.

Table 1: Treatment and outcome characteristics of Visceral Leishmaniasis patients, Uganda, 2019–2024

Treatment characteristics	Frequency	Percent
**Illness episode**		
Index episode	898	92
Recurrent episode	74	8
**Duration of treatment**		
≤2 weeks	227	23
>2	745	77
**Treatment regimen**		
AmBisome (2nd line)	229	24
S.S.G/PM (1st line)	743	76
**Treatment outcome**		
Completed/Failed	1	0
Completed/cured	957	98
Defaulted	2	0
Died	12	1

Case fatalities and associated factors among Visceral Leishmaniasis patients, Uganda, 2019–2024

The CFR fluctuated between 0% and 2% across the years (1% in 2019, 2% in 2020 and 2021, 1% in 2022 and 0% in 2023 and 2024 (Figure 4). After adjusting for country of residence and Malaria, being HIV positive was the only measured predictor associated with death (Table 2).

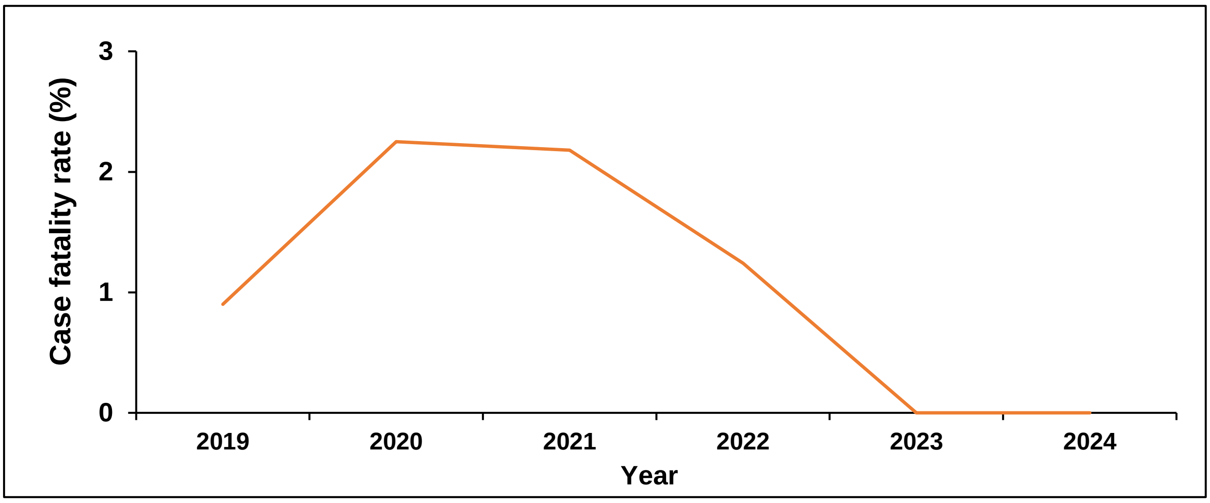

Figure 4: Case Fatality Rate of VL patients per year, 2019–2024

Table 2: Characteristics and factors associated with death among patients with Visceral Leishmaniasis, Uganda, 2019–2024

Characteristics	Dead %(n/N)	Alive %(n/N)	OR	95%CI	AOR	95%CI
**Country/Residence**						
Uganda	0.7%(4/599)	99%(595/599)	ref		ref	
Kenya	2%(8/373)	98%(365/373)	3.3	1.0-11.0	1.4	0.7-2.5
**Age**						
<5years	2%(2/129)	98%(127/129)	ref			
5-18years	0.3%(2/613)	99%(611/613)	0.2	0.03-1.5	NA	
≥19years	3.5%(8/230)	97%(222/230)	2.3	0.5-10.9	NA	
**Sex**						
Female	0.7%(2/302)	99%(300/302)	ref			
Male	1.5%(10/670)	98.5%(660/670)	2.3	0.5-10.0	NA	
**Severe Anemia**						
No	0.9%(4/460)	99%(456/460)				
Yes	1.6%(8/512)	98.4%(504/512)	1.8	0.5-6.0	NA	
**HIV status**						
Negative	0.5%(2/408)	99.55(406/408%				
Positive	5.4%(10/185)	94.6%(175/185)	12	2.5-53.0	**10**	**2.2-50.0**
**Malaria**						
Negative	1%(8/794)	99%(786/794)				
Positive	2.3%(4/175)	97.7%(171)	2.3	0.7-8.0	3.4	0.97-12.0
**Episode**						
Index	1.2%(11/898)	98.8%(887/898)				
Recurrent	1.3%(1/74)	98.7%(73/74)	1.1	0.1-8.0	NA	

OR: Odds ratio

AOR: Adjusted odds ratio

ref: Reference category

NA: not applicable, were not included in the multivariate model

Discussion

Over five and a half years there were approximately 1,000 VL patients registered at the main treatment center in Amudat which translates to an average of approximately 55 patients a month. Most patients were children, male, and over one third were from neighboring Kenya predominantly the Turkana region, while Ugandan patients were predominantly from Moroto. There was no seasonal variation in VL observed and most cases were recorded in 2022. Most patients presented with fever, night sweats and abdominal swelling and for most the illness resolved in under three months. Malaria and HIV co-infection were common and many patients

had severe anemia. The majority of HIV patients received second-line treatment regimen. Almost all patients completed treatment and were cured and the overall CFR was only approximately 1%, varying slightly across the years. Being HIV positive was associated with death.

Uganda and Kenya still account for 15 percent of the cases of Visceral leishmaniasis worldwide and it has been documented that cross-border migrations result in disease transmission between Uganda and Kenya (13). This would explain why more than one third of the VL patients in our study were from Kenya and some years even had more Kenyan patients than Ugandan. Similar studies in Uganda have reported more VL cases coming from Kenya highlighting the importance of Kenya in the persistence of VL in Uganda (15,25).It is also possible that some VL patients from Uganda are treated in Kenya which would mean that the VL counts for Uganda are an underestimate. However, this was not assessed in this study. Furthermore, Ugandan patients were predominantly from Moroto while Kenyans were predominantly from Turkana County which is Kenya's largest county, directly bordering the Karamoja region. This relationship between the most endemic regions across the two countries which facilitates continuity of VL transmission highlights the fact that elimination of VL in Uganda hinges on elimination in Kenya. This presents an opportunity for targeted collaborative interventions such as enhanced active surveillance and consequent early treatment of cases; as well as enhanced vector control activities in the high-risk border districts between the two countries. We found no seasonal variation in the number of VL cases over the years, based on the two climatic seasons experienced in the Karamoja Region of Uganda, which experiences only one rainy season from March to October (26). This is similar to a 2019 report documenting no seasonal variation of VL in Uganda and Kenya (2). However, in other non-African VL endemic countries, including India and Palestine, seasonal variations in VL incidence have been recorded. One study reviewing data for over 20 years in Palestine showed VL incidence peaking during the summer season and declining towards the winter season; while a study in India reported a peak in January which they attributed to variation in sand fly numbers likely to be positively correlated with temperature and negatively correlated with rainfall (27,28). The difference between these findings and our study may be explained by the fact that since sandflies thrive under high temperatures, and Uganda/East Africa experiences high temperatures all-year

round, it is likely that there is no significant variation in sand-fly populations across the seasons (10,29). The peak of cases in 2022 could have been a post-COVID-19 rebound effect on health seeking—resulting in more Ugandan cases seeking care following the restrictions on movement in the two years preceding.

The commonest symptoms of VL included fever, weight loss, abdominal distension due to enlarged spleen and liver, and anemia (11). All these symptoms were common among the patients in this study which likely supported the diagnosis in conjunction with the RDTs especially for the patients whose episode of VL was not their first.

Similar to malaria which is also another protozoan disease, studies have found that children are generally at greater risk of VL infection than adults in endemic areas and also suffer more complications such as severe anemia and death possibly due to their weaker immune systems (2,30–33). In keeping with this, our study found that most VL patients were children and severe anemia was found in more than half of the cases. Just as it is with anemia of malaria, anemia of VL is caused by hemolysis due to alterations in erythrocyte membrane permeability, sequestration and destruction of the erythrocytes in the enlarged spleen and hemophagocytosis of infected red blood cells (34–37).

Our study revealed more male than female VL patients and although actual incidence was not determined, studies have reported higher risk and occurrence of VL among males, with females generally accounting for smaller proportions of VL cases (15,18,27). This sex disparity is possibly due to the fact that males are more likely to engage in pastoral activities which increase their exposure to sandflies which transmit the Leishmania parasites. However, different from these findings, one study reviewing data for over 20 years in Palestine showed no sex difference in VL burden (28); suggesting similar exposures across sexes to the sand fly in this region. This could indicate that in Palestinian pastoralist communities, both males and females participate in the herding activities.

Almost all patients were diagnosed using the leishmania dipstick test, which is an RDT recommended why WHO and allows for early diagnosis and treatment, and therefore an improved prognosis compared to previous parasitological tests, which were invasive and had a longer turnaround time (20). However, being a serological

test, it can remain positive after a past infection, although for most of the patients in this study, this was an index episode of VL. For patients with a past history of VL, this test is used in conjunction with a clinical finding pathognomonic of VL, including fever and abdominal distension, to prevent unnecessarily subjecting them to toxic drugs (1,20).

Co-infection with HIV was common in our study, in keeping with similar studies that have shown significant levels of VL-HIV co-occurrence. The significantly higher HIV prevalence among VL patients in Karamoja (30%) compared to the general population (2.1%) can be attributed to an interplay of biological, behavioral, and socio-cultural factors. HIV and VL are known to have a synergistic relationship—HIV weakens the immune system, making individuals more susceptible to Leishmania infection(38,39). Additionally, risky sexual behavior often driven by poverty, conflict, or food insecurity—particularly among mobile and pastoralist communities common in Karamoja—may also contribute to HIV exposure. Many countries report cases, including Northern Ethiopia, which reportedly has the highest rate of HIV infection in VL patients, at 15–35% (38,40,41). Our study found a similar co-infection rate to that of Ethiopia. VL is also included in WHO's clinical staging of HIV as an AIDS defining condition and the two infections reinforce each other, resulting in more severe illness, increasing the risk of death (18,39,42). In keeping with this, our study showed that HIV infection was associated with death among VL patients. These findings present an opportunity for integrated programs targeting both HIV and VL in such populations. Co-infection with malaria was also observed in our study and has been documented in several studies with prevalences ranging from 4.2% to 30% (43–45).

Most patients in our study were treated with the first-line treatment regimen consisting of sodium stibogluconate plus paromomycin, although most HIV-coinfected patients were on the second-line regimen. This is in keeping with WHO and country guidelines for treatment of VL, which recommend second-line treatment for special categories including VL-HIV coinfection (20,42,46).

Mortality from VL in our study was low, almost reaching the WHO target for elimination as a public health problem (21). This was lower than reported by previous studies (2009 and 2022) in Uganda, which reported annual CFRs as high as 11% (16,18). This is indicative of how far the treatment and prognosis of VL have come

over the years and the effect of effective treatment on reducing the burden of disease (47,48).

Study limitations

Incidence of VL could not be determined because data was collected from only one treatment facility i.e. Amudat and not from Moroto and Napak. Therefore, the numbers reported here are an underestimation of the actual VL numbers in the country.

Also, given that the burden of disease was estimated passively based on existing hospital records, it might be an underestimation of actual burden considering the likelihood of subpar health seeking behaviour in the region.

Accurate determination of prevalence of co-infections was precluded by the fact that not all patients were tested for specific co-morbidities. For example, only 28 patients had results for Hepatitis B among whom 26 were positive, which is not an accurate representation of the prevalence of Hepatitis B among all VL patients.

Conclusion

We reveal high rates of cure among patients with VL, indicating progress towards elimination of VL as a public health problem. We also highlight the significance of the cross-border relationship between Uganda and Kenya in the epidemiology of disease and also underscore the prognostic importance of co-infections especially HIV.

Recommendations

We recommend targeted interventions—such as enhanced active surveillance and consequent early treatment of cases, as well as enhanced vector control activities in the high-risk border districts between Kenya and Uganda—to address cross-border vector and human-to-human transmission, as well as routine prompt screening/treatment of co-infections.

List of Abbreviations

VL: Visceral Leishmaniasis

WHO: World Health Organization

NTD: Neglected Tropical Disease

S.S.G: Sodium stibogluconate

PM: Paromomycin

CFR: Case Fatality Rate

SD: Standard Deviation

CDC: Centers for Disease Control and Prevention

RDTs: Rapid Diagnostic Tests

HIV: Human Immunodeficiency Virus

Acknowledgements

The authors thank the Vector Borne and Neglected Tropical Diseases Division of the Ministry of Health for their technical guidance.

References

1. Scarpini S, Dondi A, Totaro C, Biagi C, Melchionda F, Zama D, et al. Visceral Leishmaniasis: Epidemiology, Diagnosis, and Treatment Regimens in Different Geographical Areas with a Focus on Pediatrics. Microorganisms [Internet]. 2022 Oct [cited 2024 Jul 22];10(10). Available from: https://www.ncbi.nlm.nih.gov/pmc/articles/PMC9609364/

2. Visceral leishmaniasis [Internet]. 2015 [cited 2024 Jul 22]. Available from: https://epicentre.msf.org/en/portfolio/visceral-leishmaniasis

3. Kolaczinski JH, Worku DT, Chappuis F, Reithinger R, Kabatereine N, Onapa A, et al. Kala-azar Control, Uganda. Emerg Infect Dis. 2007 Mar;13(3):507–9.

4. Mukhtar MM, Sharief AH, El Saffi SH, Harith AE, Higazzi TB, Adam AM, et al. Detection of antibodies to *Leishmania donovani* in animals in a kala-azar endemic region in eastern Sudan: a preliminary report. Transactions of the Royal Society of Tropical Medicine and Hygiene. 2000 Jan 1;94(1):33–6.

5. Dereure J, El-Safi SH, Bucheton B, Boni M, Kheir MM, Davoust B, et al. Visceral leishmaniasis in eastern Sudan: parasite identification in humans and dogs; host-parasite relationships. Microbes and Infection. 2003 Oct 1;5(12):1103–8.

6. *Leishmania donovani*. In: Wikipedia [Internet]. 2025 [cited 2025 Jun 19]. Available from: https://en.wikipedia.org/w/index.php?title=Leishmania_donovani&oldid=1292849741

7. Pederiva MMC, Santos SM dos, Rivarola LGS, Guerreiro VJ, Lopes KS, Lima Junior MS da C, et al. Asymptomatic *Leishmania* infection in humans: A systematic review. Journal of Infection and Public Health. 2023 Feb 1;16(2):286–94.

8. Mueller YK, Kolaczinski JH, Koech T, Lokwang P, Riongoita M, Velilla E, et al. Clinical Epidemiology, Diagnosis and Treatment of Visceral Leishmaniasis in the Pokot Endemic Area of Uganda and Kenya. 2014 Jan 8 [cited 2025 Jun 19]; Available from: https://www.ajtmh.org/view/journals/tpmd/90/1/article-p33.xml

9. Molina R, Jiménez M, García-Martínez J, San Martín JV, Carrillo E, Sánchez C, et al. Role of asymptomatic and symptomatic humans as reservoirs of visceral leishmaniasis in a Mediterranean context. PLoS Negl Trop Dis. 2020 Apr 23;14(4):e0008253.

10. Phlebotomine sand flies - Factsheet for experts [Internet]. 2017 [cited 2024 Apr 13]. Available from: https://www.ecdc.europa.eu/en/disease-vectors/facts/phlebotomine-sand-flies

11. Leishmaniasis [Internet]. [cited 2024 Apr 13]. Available from: https://www.who.int/news-room/fact-sheets/detail/leishmaniasis

12. Mckinnon J. Kala-Azar in the Upper Rift Valley of Kenya. Part I. Background and Discovery of the Disease. The Journal of tropical medicine and hygiene [Internet]. 1962 [cited 2024 Jul 22]; Available from: https://www.semanticscholar.org/paper/Kala-Azar-in-the-Upper-Rift-Valley-of-Kenya.-Part-Mckinnon/c5f5ee5fd37653bf241c3443bf42c7839f4a2d6e

13. Strengthening surveillance of leishmaniasis in Uganda and Kenya through a One Health approach [Internet]. [cited 2024 Apr 13]. Available from: https://tdr.who.int/newsroom/news/item/12-12-2023-strengthening-surveillance-of-leishmaniasis-in-uganda-and-kenya-through-a-one-health-approach

14. Jones CM, Welburn SC. Leishmaniasis Beyond East Africa. Front Vet Sci [Internet]. 2021 Feb 26 [cited 2024 Jul 22];8. Available from:

https://www.frontiersin.org/journals/veterinary-science/articles/10.3389/fvets.2021.618766/full

15. Olobo-Okao J, Sagaki P. Leishmaniasis in Uganda: Historical account and a review of the literature. Pan Afr Med J. 2014 May 4;18:16.

16. Wanyana MW, Migisha R, King P, Kwesiga B, Kadobera D, Bulage L, et al. Spatial and Temporal Distribution of Visceral Leishmaniasis in Karamoja Region, Uganda: Analysis of surveillance data, 2015–2022 [Internet]. 2024 [cited 2024 Apr 13]. Available from: http://medrxiv.org/lookup/doi/10.1101/2024.02.16.24302916

17. Kolaczinski JH, Reithinger R, Worku DT, Ocheng A, Kasimiro J, Kabatereine N, et al. Risk factors of visceral leishmaniasis in East Africa: a case-control study in Pokot territory of Kenya and Uganda. Int J Epidemiol. 2008 Apr;37(2):344–52.

18. Mueller Y, Mbulamberi D, Odermatt P, Hoffmann A, Loutan L, Chappuis F. Risk factors for in-hospital mortality of visceral leishmaniasis patients in eastern Uganda. Tropical medicine & international health : TM & IH. 2009 Jun 1;14:910–7.

19. Uganda NTD Master plan 2023-2027.

20. ka_guidelines_uganda_final.doc [Internet]. [cited 2024 Jul 20]. Available from: https://view.officeapps.live.com/op/view.aspx?src=https%3A%2F%2Fwww.malariaconsortium.org%2Fuserfiles%2Ffile%2FNTD%2520Resources%2Fka_guidelines_uganda_final.doc&wdOrigin=BROWSELINK

21. Ending the neglect to attain the Sustainable Development Goals: A road map for neglected tropical diseases 2021–2030 [Internet]. [cited 2024 Feb 17]. Available from: https://www.who.int/publications-detail-redirect/9789240010352

22. Malnutrition still a challenge for children in Karamoja | UNICEF Uganda [Internet]. [cited 2024 Aug 11]. Available from: https://www.unicef.org/uganda/stories/malnutrition-still-challenge-children-karamoja

23. UPHIA-Summary-Sheet-2020.pdf [Internet]. [cited 2024 Aug 13]. Available from: https://phia.icap.columbia.edu/wp-content/uploads/2022/08/UPHIA-Summary-Sheet-2020.pdf

24. Mitra AK, Mawson AR. Neglected Tropical Diseases: Epidemiology and Global Burden. Tropical Medicine and Infectious Disease. 2017 Sep;2(3):36.

25. Kolaczinski JH, Kabatereine NB, Onapa AW, Ndyomugyenyi R, Kakembo ASL, Brooker S. Neglected tropical diseases in Uganda: the prospect and challenge of integrated control. Trends Parasitol. 2007 Oct;23(10–3):485–93.

26. World Bank Climate Change Knowledge Portal [Internet]. [cited 2024 Jul 24]. Available from: https://climateknowledgeportal.worldbank.org/

27. Jervis S, Chapman LAC, Dwivedi S, Karthick M, Das A, Le Rutte EA, et al. Variations in visceral leishmaniasis burden, mortality and the pathway to care within Bihar, India. Parasites & Vectors. 2017 Dec 7;10(1):601.

28. Amro A. Epidemiology and Spatiotemporal Analysis of Visceral Leishmaniasis in Palestine from 1990 to 2017. International Journal of Infectious Diseases. 2019 Nov 1;90.

29. Uganda - Climatology | Climate Change Knowledge Portal [Internet]. [cited 2024 Aug 11]. Available from: https://climateknowledgeportal.worldbank.org/country/uganda/climate-data-historical

30. Fact sheet about malaria [Internet]. [cited 2024 Jul 23]. Available from: https://www.who.int/news-room/fact-sheets/detail/malaria

31. Kafetzis DA. An overview of paediatric leishmaniasis. J Postgrad Med. 2003;49(1):31–8.

32. Kafetzis DA, Maltezou HC. Visceral leishmaniasis in paediatrics. Curr Opin Infect Dis. 2002 Jun;15(3):289–94.

33. Bhattacharya SK, Jha TK, Sundar S, Thakur CP, Engel J, Sindermann H, et al. Efficacy and Tolerability of Miltefosine for Childhood Visceral Leishmaniasis in India. Clinical Infectious Diseases. 2004 Jan 15;38(2):217–21.

34. Goto Y, Cheng J, Omachi S, Morimoto A. Prevalence, severity, and pathogeneses of anemia in visceral leishmaniasis. Parasitol Res. 2017 Feb;116(2):457–64.

35. Possible mechanisms of anemia during VL. Based on clinical findings,... | Download Scientific Diagram [Internet]. [cited 2024 Jul 23]. Available from: https://www.researchgate.net/figure/Possible-mechanisms-of-anemia-during-VL-Based-on-clinical-findings-possible-mechanisms_fig3_309799967

36. Castro-Gomes T, Mourão LC, Melo GC, Monteiro WM, Lacerda MVG, Braga ÉM. Potential Immune Mechanisms Associated with Anemia in Plasmodium vivax Malaria: a Puzzling Question. Infection and Immunity. 2014 Sep 10;82(10):3990–4000.

37. Anemia due to Malaria - HemoCue [Internet]. [cited 2024 Jul 23]. Available from: https://www.hemocue.com/en/health-areas/anemia/anemia-due-to-malaria

38. Visceral leishmaniasis and HIV coinfection: WHO publishes new guideline with region-specific treatment recommendations [Internet]. [cited 2024 Jul 23]. Available from: https://www.who.int/news/item/08-06-2022-visceral-leishmaniasis-and-HIV-coinfection-WHO-publishes-new-guideline-with-region-specific-treatment-recommendations

39. Visceral leishmaniasis and HIV in Tigray, Ethiopia - Lyons - 2003 - Tropical Medicine & International Health - Wiley Online Library [Internet]. [cited 2024 Jul 23]. Available from: https://onlinelibrary.wiley.com/doi/full/10.1046/j.1365-3156.2003.01088.x

40. Mohammed HB, Ali AA, Idriss MI, Gasmelseid KM, Yousif MM, Ibrahem AA, et al. Prevalence of Hepatitis B, Hepatitis C, HIV and Malaria Co Infection among Patients Infected with Visceral Leishmaniasis in Gedarif, Eastern Sudan. Global Journal of Infectious Diseases and Clinical Research. 2016 Oct 12;2(1):021–4.

41. Kantzanou M, Karalexi MA, Theodoridou K, Kostares E, Kostare G, Loka T, et al. Prevalence of visceral leishmaniasis among people with HIV: a systematic review and meta-analysis. Eur J Clin Microbiol Infect Dis. 2023 Jan 1;42(1):1–12.

42. WHO guideline for the treatment of visceral leishmaniasis in HIV co-infected patients in East Africa and South-East Asia [Internet]. [cited 2024 Jul 20]. Available from: https://www.who.int/publications/i/item/9789240048294

43. Bogaart E van den, Berkhout MMZ, Adams ER, Mens PF, Sentongo E, Mbulamberi DB, et al. Prevalence, Features and Risk Factors for Malaria Co-Infections amongst Visceral Leishmaniasis Patients from Amudat Hospital, Uganda. PLOS Neglected Tropical Diseases. 2012 Apr 10;6(4):e1617.

44. Ferede G, Diro E, Getie S, Getnet G, Takele Y, Amsalu A, et al. Visceral Leishmaniasis-Malaria Coinfection and Their Associated Factors in Patients Attending Metema Hospital, Northwest Ethiopia: Suggestion for Integrated Vector Management. Malar Res Treat. 2017;2017:6816913.

45. Ornellas-Garcia U, Cuervo P, Ribeiro-Gomes FL. Malaria and leishmaniasis: Updates on co-infection. Front Immunol. 2023 Feb 21;14:1122411.

46. VL_Guidelines_Ethiopia_2013.pdf [Internet]. [cited 2024 Jul 23]. Available from: https://www.afrikadia.org/wp-content/uploads/2018/08/VL_Guidelines_Ethiopia_2013.pdf

47. Bora D. Epidemiology of visceral leishmaniasis in India. Natl Med J India. 1999;12(2):62–8.

48. Alvar J, Den Boer M, Dagne DA. Towards the elimination of visceral leishmaniasis as a public health problem in east Africa: reflections on an enhanced control strategy and a call for action. The Lancet Global Health. 2021 Dec;9(12):e1763–9.
