# Supplementary material for: Characteristics, treatment outcomes and factors associated with death among patients with Visceral Leishmaniasis, Uganda, 2019–2024
Source: PLoS Negl Trop Dis. 2026 Feb 26;20(2):e0014032. doi: 10.1371/journal.pntd.0014032 (PMC12956114; doi:10.1371/journal.pntd.0014032)
Supplement: Attachment — Submitted filename: Response to Reviewers.docx [file pntd.0014032.s003.docx]

We sincerely thank the editors and reviewers for their thorough evaluation of our manuscript and for the constructive feedback provided. We greatly appreciate the time and expertise invested in strengthening the clarity, methodological rigor, and overall quality of our work. In this document, we address each comment point-by-point, indicating the corresponding revisions made in the manuscript. We believe these changes have substantially improved the scientific robustness and presentation of the study, and we are grateful for the opportunity to revise and resubmit our work.

**Notes:**
1. For Clarity, the reviewers’ comments have been highlighted in yellow while the authors’ responses have been highlighted in green.

2. The line numbers recorded in the authors’ response are based on the ‘revised manuscript with tracked changes’.

**Editors’ Comments:**

**Additional Editor Comments:**

The authors must describe the study design in the methods section. The figures in the results needs resolution (Figure 1) and for the rest of the figures, the legends/labels in the figures must have uniformity with text font and theirs sizes throughout.

**Authors Response 1:** I have replaced Figure 1 with a map with better resolution and uniformized the legends and labels in all the other figures.


**Journal Requirements:**

1) Please ensure that the CRediT author contributions listed for every co-author are completed accurately and in full.
At this stage, the following Authors/Authors require contributions: Benigna Gabriela Namara, Ivan Ankunda, Richard Migisha, Benon Kwesiga, Lilian Bulage, Sandra Nabatanzi, Alex Riolexus Ario, Alfred Riolexus Mubangizi, and Daniel Riolexus Kadobera. Please ensure that the full contributions of each author are acknowledged in the "Add/Edit/Remove Authors" section of our submission form.
The list of CRediT author contributions may be found here: <https://journals.plos.org/plosntds/s/authorship#loc-author-contributions>

**Authors Response 2:** This has been addressed in the resubmission.

2) Please upload all main figures as separate Figure files in .tif or .eps format. For more information about how to convert and format your figure files please see our guidelines: 
<https://journals.plos.org/plosntds/s/figures>

**Authors Response 3:** This has been addressed in the resubmission.

3) Some material included in your submission may be copyrighted. According to PLOSu2019s copyright policy, authors who use figures or other material (e.g., graphics, clipart, maps) from another author or copyright holder must demonstrate or obtain permission to publish this material under the Creative Commons Attribution 4.0 International (CC BY 4.0) License used by PLOS journals. Please closely review the details of PLOSu2019s copyright requirements here: PLOS Licenses and Copyright. If you need to request permissions from a copyright holder, you may use PLOS's Copyright Content Permission form.
Please respond directly to this email and provide any known details concerning your material's license terms and permissions required for reuse, even if you have not yet obtained copyright permissions or are unsure of your material's copyright compatibility. Once you have responded and addressed all other outstanding technical requirements, you may resubmit your manuscript within Editorial Manager. 
Potential Copyright Issues:

- Figure 1. Please (a) provide a direct link to the base layer of the map (i.e., the country or region border shape) and ensure this is also included in the figure legend; and (b) provide a link to the terms of use / license information for the base layer image or shapefile. We cannot publish proprietary or copyrighted maps (e.g. Google Maps, Mapquest) and the terms of use for your map base layer must be compatible with our CC BY 4.0 license.
Note: if you created the map in a software program like R or ArcGIS, please locate and indicate the source of the basemap shapefile onto which data has been plotted.
If your map was obtained from a copyrighted source please amend the figure so that the base map used is from an openly available source. Alternatively, please provide explicit written permission from the copyright holder granting you the right to publish the material under our CC BY 4.0 license.
If you are unsure whether you can use a map or not, please do reach out and we will be able to help you. The following websites are good examples of where you can source open access or public domain maps:
* U.S. Geological Survey (USGS) - All maps are in the public domain. ([http://www.usgs.gov](http://www.usgs.gov/))
* PlaniGlobe - All maps are published under a Creative Commons license so please cite u201cPlaniGlobe, [http://www.planiglobe.com](http://www.planiglobe.com/), CC BY 2.0u201d in the image credit after the caption. (<http://www.planiglobe.com/?lang=enl>)
* Natural Earth - All maps are public domain. (<http://www.naturalearthdata.com/about/terms-of-use/>).

**Authors Response 4:** We thank the reviewer for raising this important point. We confirm that Figure 1 was entirely generated by the authors in QGIS (version 3.10.2) and is not reproduced from any copyrighted source. The basemap was created using publicly available administrative boundary shapefiles from the Uganda Bureau of Statistics (UBOS), accessed through the UN OCHA Humanitarian Data Exchange (HDX): [https://data.humdata.org/dataset/cod-ab-uga](https://data.humdata.org/dataset/cod-ab-uga)

The dataset is distributed under a Creative Commons Attribution for Intergovernmental Organisations (CC BY-IGO**)** license, which permits reuse with proper attribution. No proprietary or copyrighted basemaps (e.g., Google Maps, MapQuest) or imagery were used; only the openly licensed vector boundary data were imported and styled within QGIS.

To ensure complete transparency, we have added the following statement to the figure legend:
***“Map created in QGIS 3.10.2 using publicly available administrative boundary shapefiles from the Uganda Bureau of Statistics (UBOS), accessed via the Humanitarian Data Exchange:*** <https://data.humdata.org/dataset/cod-ab-uga>***).”***

4) In the online submission form, you indicated that "The datasets upon which our findings are based belong to the Uganda Public Health Fellowship Program (PHFP) Ministry of Health and are not publicly available,but can be availed upon reasonable request from the corresponding author with permission from the Uganda Public Health Fellowship Program.". All PLOS journals now require all data underlying the findings described in their manuscript to be freely available to other researchers, either
1. In a public repository
2. Within the manuscript itself
3. Uploaded as supplementary information.
This policy applies to all data except where public deposition would breach compliance with the protocol approved by your research ethics board. If your data cannot be made publicly available for ethical or legal reasons (e.g., public availability would compromise patient privacy), please explain your reasons by return email and your exemption request will be escalated to the editor for approval. Your exemption request will be handled independently and will not hold up the peer review process, but will need to be resolved should your manuscript be accepted for publication. One of the Editorial team will then be in touch if there are any issues.

**Authors Response 5:** With the appropriate permissions, the data set was uploaded during the original submission under the file name **“ Epi-study_VL_CLEAN_No IDs”** .

5) Please amend your detailed Financial Disclosure statement. This is published with the article. It must therefore be completed in full sentences and contain the exact wording you wish to be published.
State the initials, alongside each funding source, of each author to receive each grant. For example: "This work was supported by the National Institutes of Health (####### to AM; ###### to CJ) and the National Science Foundation (###### to AM).".
If you did not receive any funding for this study, please simply state: u201cThe authors received no specific funding for this work.u201d

**Authors Response 6:** This has been addressed in the resubmission.

**Reviewers' Comments:**

Reviewer's Responses to Questions

**Key Review Criteria Required for Acceptance?**

As you describe the new analyses required for acceptance, please consider the following:

**Methods:**

-Are the objectives of the study clearly articulated with a clear testable hypothesis stated?

-Is the study design appropriate to address the stated objectives?

-Is the population clearly described and appropriate for the hypothesis being tested?

-Is the sample size sufficient to ensure adequate power to address the hypothesis being tested?

-Were correct statistical analysis used to support conclusions?

-Are there concerns about ethical or regulatory requirements being met?

Reviewer #1: Need for study (Background) needs to be specified more clearly.
Methodology is clear.

**Author’s response 7:** I have added a paragraph in the background to address this. It states:

“Despite decades of VL control efforts in Uganda, the true clinical burden, treatment outcomes, and mortality risk factors remain poorly documented, particularly in the high-transmission Karamoja region. Existing national data rely almost entirely on aggregated DHIS2 reports, which are limited by under-diagnosis, variable reporting quality, and lack of patient-level detail necessary to guide elimination efforts. Moreover, the most recent published analyses date back to 2014 and 2022, neither of which characterized individual patients nor examined clinical outcomes or predictors of death, leaving critical evidence gaps in a context where Uganda aims to reduce VL case fatality to <1% by 2030. Given the region’s unique epidemiologic profile—including intense cross-border movement with highly endemic Kenyan districts, high malnutrition rates, and the increasing importance of HIV co-infection—current, granular data are urgently needed to assess progress toward national targets and identify modifiable factors that influence patient outcomes. By analyzing individual-level data from the country’s main treatment center over a five-year period, this study fills a key gap in understanding who is affected, how patients are currently faring under routine program conditions, and what factors continue to drive mortality, thereby providing actionable evidence to strengthen surveillance, case management, and cross-border VL control strategies.” **Lines 148-164**

Reviewer #2: #Reviewer Comments
I appreciate the opportunity to review this manuscript, which addresses a topic of significant relevance to Uganda and the African continent. Below I provide specific considerations and suggestions that may strengthen the work.

#Abstract

Comment - It may be more effective to begin by reaffirming that “visceral leishmaniasis, a neglected tropical disease, continues to affect several countries, including Uganda.”

**Author’s response 8:** Thank you for this comment. I have revised the opening statement to say:

“Visceral leishmaniasis (VL), a neglected tropical disease, continues to affect several countries worldwide, including Uganda, where it remains a significant public health concern in the Karamoja region.”**Lines 16-18.**

Comment - Consider highlighting the underlying reasons for the persistence of this disease globally and in East Africa.

**Author’s response 9:** Thank you for this comment.I have added a statement that says:

“Globally and within East Africa, VL persists due to a combination of ecological suitability for sandfly vectors, chronic underdiagnosis, limited access to care in remote and pastoralist communities, high levels of malnutrition and poverty, and cross-border population movement that sustains transmission.” **Lines 19-23**

Comment - In the Methods section, it is important to specify the study design (which appears to be observational and cross-sectional). This should also be made explicit in the main text of the manuscript.
**Author’s response 10:** Thank you for this comment. I have revised the method’s opening statement to say:

“We conducted a retrospective observational review of patient records from 2019–2024 at the main VL treatment center in Amudat District, Uganda, abstracting socio-demographic, clinical, treatment, and outcome data.” **Lines 28-30 in the abstract and lines 169-171 in the manuscript body.**

Comment- In the Results section, please review the expression “(80%, [259/322).” The presentation of these results should be standardized throughout the manuscript.
**Author’s response 11:** Thank you for this comment. I have revised the statement to say:

“The highest number of cases (322) were recorded in 2022, with Ugandans making up 80% of all patients that year (259/322), unlike 2019–2021 when Kenyan patients predominated.”**Lines 38-41**

#Theoretical Foundation
Comment - It would strengthen the contextualization to cite elimination programs conducted in East Africa and in India, as these provide relevant comparative experiences.
 **Author’s response 12:** Thank you for this comment. In the background I have added a paragraph to this effect that states:

“Efforts to eliminate VL as a public health problem have been undertaken in several high-burden settings, offering important lessons for Uganda. In East Africa, the regional VL elimination strategy—led by Kenya, Ethiopia, Sudan, South Sudan, and Uganda—emphasizes early case detection, expanded access to effective treatment regimens, and strengthening cross-border coordination to interrupt transmission. Similarly, the VL elimination initiative in the Indian subcontinent (India, Nepal, and Bangladesh) achieved dramatic reductions in incidence through sustained vector control, decentralized diagnosis with rapid tests, community-based surveillance, and investment in strong referral systems. These programs demonstrate that elimination is feasible when supported by systematic surveillance, early treatment, and coordinated regional action, all of which are highly relevant to Uganda’s elimination targets.” **Lines 136-147**

**#Methodology**

Comment - Figure 1 is relevant and informative, but a higher-resolution version would be beneficial.
**Authors response 13:** Thank you for this. I have changed the figure to one with better resolution.

Comment - In Figure 2, consider harmonizing the font used for the numbers with that of the main text for consistency.
**Authors response 14:** Thank you for this. I have changed all the fonts to Arial.

Comment - The study is limited to a single center (Amudat Hospital), which reduces its national representativeness. It is important to clarify whether Amudat is the only treatment center or whether this is a convenience sample.

**Authors response 15:** Thank you for this comment. This has been addressed in both the methods and limitations sections as follows:

**Methods:** “Amudat hospital was selected because it is the main VL treatment center and has been operational since 2000, while the newer centers in Moroto and Napak have only been operational since 2023. Consequently, the study reflects a convenience sample of patients presenting at Amudat rather than a nationally representative population.” **Lines 175-178**

**Limitations:** “The incidence of VL could not be determined as data were collected solely from Amudat Hospital and not from the newer treatment centers in Moroto and Napak. Consequently,, the numbers reported likely underestimate the actual VL numbers in the country. Although Amudat Hospital is the main and longest-standing VL treatment center in Uganda, the study sample reflects a convenience sample rather than a nationally representative dataset. During most of the study period, Amudat functioned as the primary center for VL diagnosis while the other centers only became operational in the last two years. As a result, the findings predominantly represent patients who sought care at Amudat and may not capture VL patterns in districts where individuals accessed services across the border in Kenya or at the newer Ugandan treatment centers.” **Lines 415-426**

Comment - Please provide a stronger justification for the choice of study period and for the exclusion of other reference centers. Was this due to operational constraints (e.g., data availability), or were there other specific reasons?
**Author’s response 16:** I have clarified this in the methods section which now states:

”The study period was selected to capture the most recent five years of patient data, providing a contemporary view of VL trends while remaining feasible within the available time and resources for data collection and analysis.” **Lines 172-175**

Comment - The manuscript does not mention approval by a Human Research Ethics Committee. This is a critical omission, particularly given the use of patient medical records. Information about ethical clearance and patient confidentiality protections must be included.
 **Author’s response 17:** Thank you. I had removed most of the declarations and supplementary material including ethical considerations from the main body of the manuscript, as required by the journal during submission. The original document does have the following ethical statement:

 “**Ethics approval and consent to participate:** Administrative clearance to conduct this study was obtained from the NTD control program and the Amudat hospital administration. The study used routinely collected data from patients records and no participant enrollment was involved. The office of the Center for Global Health, US Center for Disease Control and Prevention determined that this activity was not human subject research and with its primary intent being for public health practice or disease control. This activity was reviewed by CDC and was conducted consistent with applicable federal law and CDC policy. § §See e.g., 45 C.F.R. part 46, 21 C.F.R. part 56; 42 U.S.C. §241(d); 5 U.S.C. §552a; 44 U.S.C. §3501 et seq.”

**Results**


-Does the analysis presented match the analysis plan?

-Are the results clearly and completely presented?

-Are the figures (Tables, Images) of sufficient quality for clarity?

Reviewer #1: No comments for authors.

Reviewer #2: #Results and Discussion
- The results are well described and supported by clear quantitative evidence;

Comment - The statement about a “post-COVID-19 rebound effect” should be qualified as a possibility rather than presented as fact, unless supporting references are provided;

**Author’s response 18:** Thank you for this comment. The statement has been revised to say:

“The peak of cases in 2022 could have been a post-COVID-19 rebound effect on health seeking behaviour as movement restrictions and service disruptions in the preceeding years could have temporarily suppressed care-seeking and reporting. However, this interpretation should be considered cautiously because we did not directly assess the influence of COVID-19 on VL service utilization.” **Lines 326-330.**

Comment - Some paragraphs are too long (lines 265-297; 331-348), making them difficult to read;
**Author’s response 19:** This has been addressed. The paragraphs have been shortened. Thank you for this.

Comment - A valuable addition would be to expand the discussion on how lessons learned from this study could inform strategies in other countries of the Global South facing similar challenges. Making comparisons with other countries in the Global South would elevate the work to a broader level and context.
**Author’s response 20a:** I have addressed this by adding a few few paragraphs in **Lines 397-413** which now state:

“The findings from this study have important implications for other VL‑affected countries in the Global South, many of which face similar challenges of cross‑border transmission, fragile health systems, limited diagnostic capacity, and high burdens of co‑infections such as HIV and malaria(51). For instance, VL–HIV co‑infection remains a major driver of mortality, particularly in countries such as Ethiopia, where HIV-positive VL patients have significantly worse treatment outcomes and higher relapse rates(52).

Likewise, cross-border mobility has been documented as a key barrier to VL elimination. In East Africa, governments of Kenya, Ethiopia, Sudan, and other countries are increasingly prioritizing regional coordination for surveillance and treatment(51).In South Asia, persistent VL transmission in the India–Nepal–Bangladesh corridor, including new foci in border districts, highlights how population movement and cross-border infection risk complicate elimination efforts(53).

The high cure rates and low CFR in Amudat demonstrate the value of sustained availability of effective treatment, early diagnosis using RDTs, and programmatic emphasis on comorbidity management. These lessons are broadly applicable to countries with similar pastoralist or mobile populations, semi-arid ecology favorable to sandflies, and decentralized surveillance systems.”

**Author’s response 20b:** I have also added a recommendation that states:

“In addition, strengthening border-region surveillance, harmonizing treatment protocols across neighboring countries, and integrating VL services within HIV and malaria programs may therefore accelerate progress toward VL elimination in Uganda and across multiple regions of the Global South.” **Lines 453-456**

**Conclusions:**

-Are the conclusions supported by the data presented?

-Are the limitations of analysis clearly described?

-Do the authors discuss how these data can be helpful to advance our understanding of the topic under study?

-Is public health relevance addressed?

Reviewer #1: No comments for authors.

Reviewer #2: #Conclusions
The authors could expand their conclusions by including considerations for interpreting the results. However, the sample used in the analysis needs improvement.

**Author’s response 21:** Thank you for this insightful comment. We have expanded the Conclusion section to include interpretive considerations, specifically discussing how the findings should be understood in light of the study’s sampling limitations. We have also clarified that the sample was restricted to a single treatment center and we outline the implications for representativeness and generalizability. The revised conclusion now acknowledges these issues and clearly communicates the appropriate scope of interpretation **in Lines 435-446** which now state:

“Our findings reveal high cure rates and low case fatality rates among patients treated for VL in Uganda, suggesting meaningful progress towards elimination of VL as a public health problem. We also highlight the significance of the cross-border relationship between Uganda and Kenya in the epidemiology of disease and also underscore the prognostic importance of co-infections especially HIV.

However, these findings should be interpreted with caution because the sample used reflects patients treated at Amudat Hospital,but does not fully represent all VL cases nationally, particularly those managed at the newer centers in Moroto and Napak or across the border in Kenya, which limits generalizability.

Despite these limitations, the study provides critical patient-level evidence that can guide targeted interventions, inform VL program strengthening, and support ongoing efforts toward VL elimination in Uganda and the broader East African region.”

**Editorial and Data Presentation Modifications?**

Use this section for editorial suggestions as well as relatively minor modifications of existing data that would enhance clarity. If the only modifications needed are minor and/or editorial, you may wish to recommend “Minor Revision” or “Accept”.

Reviewer #1: Minor Revision
Table 1 and Introduction.
**Author’s response 22:** These have been addressed in Author’s responses **24 and 25.**

Reviewer #2: #General comment
The manuscript addresses an important public health issue and presents useful findings. Incorporating the clarifications above (particularly regarding methodology, ethical approval, and contextual discussion) would improve the rigor and impact of the work.

**Author’s response 23:** This has been addressed in all the relevant sections as described in author’s responses **10, 15, 16, 17 and 20.**

**Summary and General Comments:**

Use this section to provide overall comments, discuss strengths/weaknesses of the study, novelty, significance, general execution and scholarship. You may also include additional comments for the author, including concerns about dual publication, research ethics, or publication ethics. If requesting major revision, please articulate the new experiments that are needed.

Reviewer #1: PEER REVIEW REPORT PNTD-D-25-01017

Summary
This retrospective study examines Visceral Leishmaniasis cases in Uganda from 2019 to 2024, analysing patient characteristics, treatment outcomes, and mortality factors to evaluate progress toward World Health Organization elimination targets. Visceral Leishmaniasis, commonly known as kala-azar, represents a significant neglected tropical disease burden in Uganda's semi-arid Karamoja region, which borders Kenya where the disease is endemic. The research utilized secondary data from patient files at the primary treatment facility in Amudat District, employing logistic regression analysis to identify mortality-associated factors.
The analysis encompassed 972 patient records, revealing that most patients were male (69%) and under 18 years of age (76%). Notably, 38% of cases originated from neighbouring Kenya, while 72% of Ugandan patients came from Moroto District. Case numbers peaked at 322 in 2022, with a marked shift toward more Ugandan patients compared to earlier years when Kenyan cases predominated. Clinical manifestations commonly included fever (98%), night sweats (77%), and abdominal swelling (72%). Severe anaemia affected 53% of patients, while co-infections were frequent, with malaria present in 18% and HIV in 31% of cases.
Treatment outcomes demonstrated remarkable success, with 98% of patients achieving cure rates using predominantly first-line therapy regimens. The overall case fatality rate remained low at 1%, declining from 2% in 2020-2021 to below 1% in 2023-2024. HIV co-infection emerged as the strongest mortality predictor, with an adjusted odds ratio of 10. These findings indicate substantial progress toward elimination goals through effective treatment protocols and high cure rates. However, persistent cross-border transmission challenges and the critical impact of HIV co-infection underscore the need for enhanced surveillance, early intervention strategies, and integrated public health approaches targeting high-risk border areas to achieve complete elimination objectives.

Remarks
Great work Authors.
I am really sorry for my late review.
Introduction- Towards the end of the background, the motive behind this study should be specifically mentioned (Need for study/ Rationale).
Table 1- Treatment outcome percentages are not totalling to 100.
Thank You

**Author’s response 24:** I have added a paragraph in the background to address this. It states:

 “Despite decades of VL control efforts in Uganda, the true clinical burden, treatment outcomes, and mortality risk factors remain poorly documented, particularly in the high-transmission Karamoja region. Existing national data rely almost entirely on aggregated DHIS2 reports, which are limited by under-diagnosis, variable reporting quality, and lack of patient-level detail necessary to guide elimination efforts. Moreover, the most recent published analyses date back to 2014 and 2022, neither of which characterized individual patients nor examined clinical outcomes or predictors of death, leaving critical evidence gaps in a context where Uganda aims to reduce VL case fatality to <1% by 2030. Given the region’s unique epidemiologic profile—including intense cross-border movement with highly endemic Kenyan districts, high malnutrition rates, and the increasing importance of HIV co-infection—current, granular data are urgently needed to assess progress toward national targets and identify modifiable factors that influence patient outcomes. By analyzing individual-level data from the country’s main treatment center over a five-year period, this study fills a key gap in understanding who is affected, how patients are currently faring under routine program conditions, and what factors continue to drive mortality, thereby providing actionable evidence to strengthen surveillance, case management, and cross-border VL control strategies.” **Lines 148-164**

**Author’s response 25:** This was due to rounding off but I have revised the decimal places to ensure the 100 total.

Reviewer #2: The study has a significant methodological problem that impacts the extrapolation of results. Furthermore, the manuscript fails to mention approval by a Human Research Ethics Committee.
**Authors response 26**: This concern has been addressed in all the relevant sections as described in author’s responses **10, 15, 16, 17 and 20.**

PLOS authors have the option to publish the peer review history of their article ([what does this mean?](https://track.editorialmanager.com/CL0/https:%2F%2Fjournals.plos.org%2Fplosntds%2Fs%2Feditorial-and-peer-review-process%23loc-peer-review-history/1/010f019a12003cf6-422cf5e5-e51a-408c-8beb-8fa8f41c70a6-000000/wI6MrwzumrcPsTw-OHsOzAL7PMhss5JyhEOo9kdag-8=234)). If published, this will include your full peer review and any attached files.


If you choose “no”, your identity will remain anonymous but your review may still be made public.


**Do you want your identity to be public for this peer review?** For information about this choice, including consent withdrawal, please see our [Privacy Policy](https://track.editorialmanager.com/CL0/https:%2F%2Fwww.plos.org%2Fprivacy-policy/1/010f019a12003cf6-422cf5e5-e51a-408c-8beb-8fa8f41c70a6-000000/uIwj7W4OPtOMUzuMpVQLjUwnIxM9mAaTS-u2CMXk4VQ=234).

Reviewer #1: **Yes:** DR. DENNY MATHEW JOHN
Department of Community Medicine
Kerala Medical College Hospital
Palakkad, Kerala, India

Reviewer #2: No

[NOTE: If reviewer comments were submitted as an attachment file, they will be attached to this email and accessible via the submission site. Please log into your account, locate the manuscript record, and check for the action link "View Attachments". If this link does not appear, there are no attachment files.]

**Figure resubmission:**

While revising your submission, we strongly recommend that you use PLOS’s NAAS tool (<https://ngplosjournals.pagemajik.ai/artanalysis>) to test your figure files. NAAS can convert your figure files to the TIFF file type and meet basic requirements (such as print size, resolution), or provide you with a report on issues that do not meet our requirements and that NAAS cannot fix.

After uploading your figures to PLOS’s NAAS tool - <https://ngplosjournals.pagemajik.ai/artanalysis>, NAAS will process the files provided and display the results in the "Uploaded Files" section of the page as the processing is complete. If the uploaded figures meet our requirements (or NAAS is able to fix the files to meet our requirements), the figure will be marked as "fixed" above. If NAAS is unable to fix the files, a red "failed" label will appear above. When NAAS has confirmed that the figure files meet our requirements, please download the file via the download option, and include these NAAS processed figure files when submitting your revised manuscript.

**Author’s response 27:** This is noted and appreciated. Thank you. The figure files have been tested using the NAAS tool as advised.

**Reproducibility:**

To enhance the reproducibility of your results, we recommend that authors of applicable studies deposit laboratory protocols in [protocols.io](http://protocols.io/), where a protocol can be assigned its own identifier (DOI) such that it can be cited independently in the future. Additionally, PLOS ONE offers an option to publish peer-reviewed clinical study protocols. Read more information on sharing protocols at <https://plos.org/protocols?utm_medium=editorial-email&utm_source=authorletters&utm_campaign=protocols>

**Author’s response 28:** We appreciate the suggestion. However, this study did not involve laboratory procedures, as it was based entirely on retrospective analysis of routinely collected patient data. Therefore, depositing laboratory protocols or publishing a clinical study protocol is not applicable in this context.
